# A survey of caregiver burden in those providing informal care for patients with schizophrenia or bipolar disorder with agitation: results from a European study

**DOI:** 10.1186/s12991-018-0178-2

**Published:** 2018-02-10

**Authors:** Sophee Blanthorn-Hazell, Alfredo Gracia, Jenna Roberts, Anca Boldeanu, Davneet Judge

**Affiliations:** 1 Adelphi Real World, Manchester, UK; 2 Patient Advocacy, Ferrer, Barcelona, Spain; 3 Scientific Department, Ferrer, Barcelona, Spain

**Keywords:** Schizophrenia, Bipolar disorder, Agitation, Caregiver, Burden

## Abstract

**Background:**

Agitation is a common feature of bipolar disorder and schizophrenia. Previous research indicates that specific symptoms impact caregiver burden in these conditions, but the impact of agitation on caregiver experience is poorly understood. The aim of this study was to characterise caregiver burden in providers of informal care for patients with bipolar disorder and schizophrenia who experience agitation.

**Methods:**

In total, 297 matched patient and caregiver surveys were collected across the UK, Germany and Spain between October 2016 and January 2017. To be eligible, caregivers needed to provide informal care to a patient with a diagnosis of bipolar disorder or schizophrenia with agitation managed in a community setting and participating in the patient survey. The caregiver survey captured information on demographics and their role in managing the patient’s agitation. Caregiver burden was assessed using the Involvement Evaluation Questionnaire. Descriptive analysis was conducted.

**Results:**

Caregivers provided 38.3 h (SD ± 40.34) a week of support to the patient with 20% providing 50 h or more. Most caregivers reported that they recognised an episode of agitation all of the time (44%, *n* = 130) or sometimes (40%, *n* = 119). Verbal de-escalation techniques (talking (80%, *n* = 239) and soothing (73%, *n* = 218) were the most commonly reported strategies used by caregivers during an episode of agitation; 14% (*n* = 43) reported resorting to physically restraining the patient. Caregivers supervised rescue medication administration regularly (41%, *n* = 69) or occasionally (49%, *n* = 82). Mean Involvement Evaluation Questionnaire score was 32.2 (± 15.27), equivalent to 28.4 (± 13.56) in Germany, 35.6 (± 16.55) in Spain and 33.3 (± 15.15) in the UK. Involvement Evaluation Questionnaire scores were higher for caregivers who reported hostile (41.7 ± 17.07) lack of control (40.3 ± 16.35) and violent (39.5 ± 16.40) patient behaviours when agitated. Over excitement (31.8 ± 15.05), restless (32.6 ± 14.77) and tense (32.9 ± 15.64) behaviours were associated with a lower Involvement Evaluation Questionnaire score.

**Conclusions:**

Caregivers are active participants in the recognition and management of agitation episodes. The substantial burden reported by these caregivers is impacted by factors including the number of hours of care provided, patient behaviours and country. These may be viable targets for effective interventions to reduce caregiver burden.

## Background

Agitation is a syndrome commonly experienced by patients suffering with psychiatric conditions, such as bipolar disorder or schizophrenia. Previous definitions have included restlessness with semi-purposeful or excessive activity, irritability and an unstable clinical course [1] or excessive motor activity, verbal aggression or physical aggression with behaviours in line with emotional distress [2]. In some cases, it may progress to aggression or violence; however, this is not a defining aspect and the exact association between agitation and violence is unclear [2, 3]. Recent literature recommends verbal de-escalation and changes to patient environment as preferred first line treatment options followed by pharmacological intervention, with restraint as a last resort [4]. The impact of agitation on the healthcare system has been previously described; a 2013 analysis of Spanish Acute Impatient admissions identified that agitation increased the cost of an admission between €282 and €822, dependant on the interventions required to manage the episode [5]. However, the impact of agitation on costs to the healthcare system for community dwelling patients is not well known.

Although agitation states from mild to severe and their characterization have been previously described, there has been a failure to reach a consensus on the definition of agitation [6, 7] which has been recognised as one factor underlying the lack of epidemiological data in this area [8]. Previous studies that have reported on the prevalence of agitation focus on emergency hospital or clinic attendance; a European study found that 4.6% of psychiatric emergencies were attributed to acute agitation, of which half of the patients had a diagnosis of schizophrenia and a quarter had a diagnosis of bipolar disorder [9]. Conversely, a US study found that 52% of psychiatric emergencies included agitation to some degree; [10] the discrepancy between the two studies is likely a result of differential definitions and an under-diagnosis of mild or moderate episodes [9]. The data from these studies indicate that agitation is observed in psychiatric emergencies, but do not provide insight into the day-to-day burden of agitation in the community.

The deinstitutionalization of metal healthcare in the last 50 years to community based care [11] has shown both economic and patient centred benefits [11–13]. However, the relocation of patients back into the home, in combination with insufficient provision of services, has increased the role of family and friends as care providers [14]. This increased burden on the patient’s family and friends often results in a negative impact on the physical and psychological health of caregivers themselves [15]. Furthermore, informal caregivers of patients with mental health conditions experience a higher burden in comparison to those providing informal care for patients with chronic medical illness [16].

The emotional, physical, financial and social burden associated with caregiving for those with schizophrenia and bipolar disorder has been previously reported [17–21]. This may partly be attributable to the high levels of care psychiatric patients require; previous research reported that 44% of informal caregivers for patients with schizophrenia and 65% of caregivers for patients with bipolar disorder have over 32 h of contact time with the patient a week [22, 23]. The impact of specific patient behaviours, as a result of particular psychiatric diseases, has been previously explored against the experience of caregivers. Schizophrenia symptoms termed as ‘positive’ (e.g. hallucinations) have been shown to result in a higher caregiver burden than ‘negative’ behaviours [23]. Additionally, 89% of bipolar patient caregivers reported moderate or high burden as a result of disease-specific behaviours [21]. For example, patients exhibiting symptoms such as hyperactivity and irritability were reported by caregivers as the most distressing behaviours [24]. Whilst some similarities can be drawn between the symptoms included in these studies and those experienced during an episode of agitation, there is limited research on the direct impact of agitation on caregiver burden. By meeting this evidence gap, the awareness and understanding of agitation would be improved within the community setting in addition to insight into the impact of agitation on the respective caregiver’s quality of life. The following study has been conducted to understand the experiences of informal caregivers for patients with schizophrenia or bipolar disorder who experience episodes of agitation.

## Methods

### Study population

The study was a cross-sectional patient and matched caregiver survey of patients diagnosed with bipolar disorder or schizophrenia in the UK, Germany and Spain between October 2016 and January 2017. To be eligible to participate, patients were required to be 18 years or older, currently treated in a community-based setting opposed to an inpatient setting, have schizophrenia or bipolar disorder and must have experienced at least one episode of agitation within the 12 months prior to completing the survey for which they had seen a healthcare professional. Patients who were currently involved in a clinical trial were not eligible to participate. The following definition of agitation was provided to ensure a consistent understanding ‘When people experience an episode of agitation they may feel noticeably more tense, restless, wound up, uneasy or short-tempered than usual. Some people talk a lot more than they would usually or find it difficult to keep still. Sometimes agitation leads to violent or aggressive behaviour but this isn’t always the case. An episode of agitation will subside after a while’. Patients were selected through their treating physician or patient support group and invited their informal caregiver to participate.

To be eligible to participate, caregivers were required to be 18 years or older and provide informal care to a patient participating in the study. Caregivers who were paid to provide care to the patients were ineligible. Results of the patient survey are reported elsewhere.

### Survey instrument

Patients and caregivers completed surveys either online or via pen and paper, and matching patient-caregiver surveys were linked for relevant responses. Both surveys took approximately 15 min to complete and captured information on; respondent demographics, disease characteristics, burden of agitation to the respondent, awareness of agitation episodes, patient symptoms of agitation, coping strategies for agitation episodes, and treatment satisfaction. The patient survey captured specific information on disease severity, number of episodes in the last 12 months that required healthcare professional support or hospitalisation and current treatment. The caregiver survey also captured questions on their role in managing the patient’s agitation, and the Involvement Evaluation Questionnaire (IEQ) “Caregiving consequences of psychiatric disorders” module was also included. This is a validated 31-item questionnaire designed to be completed by caregivers of patients with mental health disorders. 27-items of the questionnaire are divided into four subscales: tension (9-items), supervision (6-items), worrying (6-items) and urging (8-items). Two of the items are used in more than one subscale. A higher score correlates with a greater burden on the caregiver; the maximum score is 108, across all domains. A full description of the IEQ and how it is analysed has been previously reported [25].

### Statistical analyses

Descriptive analysis was performed on the data with no formal hypothesis tested. The output was dependant on the type of variable described; for numeric variables, the respondent base, mean and range (minimum and maximum values); for categorical variables the number and percentage of subjects in each category were reported. A two-sample *z* test was conducted to determine a *p* value and indicate the probability of there being a difference between the subgroups in the population. Missing data were removed from the specific analysis. However, participants removed from one piece of analysis are still eligible for inclusion in other analyses.

The IEQ was scored according to the validated scoring manual. All analysis was conducted using IBM^®^ SPSS^®^ Data Collection Survey Reporter Version 7.

## Results

A total of 583 patients and 297 caregivers were surveyed across 3 countries: Germany (*n* = 202, *n* = 99), Spain (*n* = 200, *n* = 97) and the UK (*n* = 181, *n* = 101), respectively. In total, 51% (*n* = 297) of the patient surveys had matched caregiver survey completed, the final sample described here is based on the matched sample of 297 patients and 297 caregivers. Caregiver and patient demographics and characteristics are summarised in Tables 1, 2, respectively.

**Table 1:** Caregiver demographics and characteristics

	Caregiver (*n* = 297)
Mean age (SD)	44.8 (13.13)
Gender (*n*, % female)	214 (72%)
Current employment status	
Working full time	95 (32%)
Working part time	95 (32%)
Student	16 (5%)
Homemaker	27 (9%)
Retired	39 (13%)
Unemployed	33 (11%)
Employment status before providing care for the patient	
Working full time	158 (53%)
Working part time	64 (22%)
Student	30 (10%)
Homemaker	18 (6%)
Retired	9 (3%)
Unemployed	27 (9%)
Change in employment status due to patients bipolar/schizophrenia	
Base (*n*)	140
Yes	68 (49%)
Relationship to patient	
Partner/spouse	114 (39%)
Parent	53 (18%)
Friend/neighbour	29 (10%)
Child	38 (13%)
Sibling	40 (14%)
Other	11 (3%)
Other family member	11 (4%)
Personal diagnosis of mental health disorder (*n*, % yes)	57 (19%)
Diagnosed mental health condition	
Base (*n*)	57
Depression/anxiety	39 (68%)
Other	25 (44%)
Mean hours of care provided a week (SD) (h)	38.3 (40.34)
< 10	29 (10%)
10–19	65 (22%)
20–29	80 (27%)
30–39	42 (14%)
40–49	23 (8%)
≥ 50	58 (20%)
Mean hours of care provided a week by other informal caregivers (SD)	14.3 (22.06)

**Table 2:** Patient demographics and characteristics

	Patients with a matched caregiver (*n* = 297)
Mean age (SD)	43.6 (13.31)
Gender (*n*, % female)	136 (46%)
Diagnosis	
Schizophrenia	138 (46%)
Bipolar	159 (54%)
Mean years since diagnosis (SD)	14.7 (10.53)
Employment status	
Working full time	35 (12%)
Working part time	40 (13%)
Not in workforce	222 (75%)
Living circumstances	
Live alone	40 (13%)
Live with partner/spouse	119 (40%)
Live with other family member	83 (28%)
Other	54 (18%)

### Role as caregiver

When asked about recognising agitation, 44% (*n* = 130) of caregivers reported that they are always able to recognise when a patient is experiencing an episode of agitation, followed by 40% (*n* = 119) reporting sometimes, 15% (*n* = 44) occasionally and only 1% (*n* = 4) never. When asked about interventions, 11% (*n* = 33) of caregivers reported that they will wait for an episode of agitation to become severe before intervening, whilst 52% will intervene for mild and moderate episodes (16%, *n* = 45 and 36%, *n* = 103) and 31% (*n* = 89) would wait until the episode was moderate-intense. The majority of caregivers reported using verbal de-escalation (talking (80%, *n* = 239), soothing (73%, *n* = 218) and encouraging (61%, *n* = 180) during episodes; a small proportion of caregivers reported resorting to physically restraining the patient during an episode of agitation (14% *n* = 43). When asked about medication, 90% of caregivers reported supervising the patient in taking their rescue medication for agitation regularly (41%, *n* = 69) or occasionally (49%, *n* = 82).

### Impact of caregiving

Caregivers provided an average of 38.3 (± 40.34) hours a week of care, with 20% (*n* = 58) providing at least 50 h of care a week (Table 1). Out of 297 caregivers, 50 had missing data resulting in a total sample of n = 247 for the IEQ analysis, which is summarised in Table 3.

**Table 3:** IEQ mean sum scores by key splits

	Base	Mean IEQ sum score (SD)
Total sample	247	32.2 (15.27)
Country		
Germany	93	28.4 (13.56)
Spain	75	35.6 (16.55)
UK	79	33.3 (15.15)
Main mental health disorder of patient		
Schizophrenia	106	31.9 (14.16)
Bipolar disorder	136	32.5 (16.34)
Don’t know	5	27.2 (5.81)
Caregiver current employment status		
Working full time	78	28.3 (14.81)
Student	15	28.6 (16.80)
Working part time	78	32.5 (14.53)
Homemaker	22	38.8 (17.04)
Unemployed	26	36.5 (15.07)
Retired	33	32.4 (14.76)
Mean hours of care provided a week		
1–10	44	24.5 (12.44)
11–20	66	27.8 (13.78)
21–30	65	33.5 (14.19)
31–40	20	37.3 (10.18)
41–50	16	34.1 (12.27)
> 50	36	43.4 (18.65)
Caregiver lives with patient		
Yes	153	33.2 (16.20)
No	94	30.5 (13.55)
Patient symptoms recognised by the caregiving during an episode of agitation		
Over excited	140	31.8 (15.05)
Tense	161	32.9 (15.64)
Hostile	51	41.7 (17.07)
Un-cooperative	46	38.3 (14.42)
A lack of control	16	40.3 (16.35)
Wound up	117	35.6 (15.28)
Fidgety	130	35.0 (15.03)
Violent	41	39.5 (16.40)
Aggressive	35	37.8 (11.61)
Irritable	134	34.1 (14.95)
Short tempered	79	38.4 (14.82)
Restless	157	32.6 (14.77)
Uneasy	155	33.1 (15.14)
Nervous	169	33.7 (15.73)
Feeling like they can’t sit still	141	34.3 (15.81)

When the IEQ was analysed by domain caregivers scored highest for ‘worrying’, totalling 44.6% of the maximum score, followed by ‘urging’ (36.8%) and ‘tension’ (23.7%). The ‘supervision’ domain scored the lowest with 17.8% of the maximum score. Caregivers in Spain scored a notably higher sum score within the ‘worrying’ domain compared to German caregivers (53.5% vs. 39.1%); no other relevant differences were observed.

## Discussion

Both schizophrenia and bipolar disorder are chronic conditions that have a direct, notable impact on the family and friends of those affected. The majority of caregivers surveyed in this real-world survey were female and family members. The data presented here show that, on average, caregivers are providing 38.3 h of care a week and 69% provided at least 20 h a week. Existing studies have shown high intensity caregiving to have a negative impact on health. Distress, anxiety and mood symptoms have been previously noted amongst informal caregivers of patients with bipolar disorder, and carers themselves are a high-risk groups for developing psychiatric disorders [26]. A prospective study identified double the amount of psychological distress in high intensity caregivers versus non-caregivers and of note, distress was higher in females and those caring for a partner or spouse [27]. However, similarities must be drawn with caution given the distinctly different patient populations included in this survey and the prospective study above, which is likely to impact burden.

The IEQ results derived from this real-world survey indeed show that caring for patients with schizophrenia or bipolar disorder with agitation has a significant impact on caregiver life. In agreement with previous studies using alternative methodologies, caregivers are affected across multiple domains [28, 29]. The values are comparable to previously reported IEQ scores in bipolar disorder, [22] and schizophrenia, [23] with the differences observed most likely attributable to the differences in populations with neither study assessing agitation as a specific subset of symptoms. Another factor may be the countries included in the survey; as seen here the IEQ sum score for respondents in Spain was 1.25 times higher than in Germany. A study comparing the family burden of caregiving for patients with schizophrenia in Germany and the UK found that burden was higher in the UK, a result attributed to the greater reliance on informal caregivers over professional care. Further research is required to determine if the country variance in IEQ scores derived from this study are a result of cultural differences, or differences in the management of these patients that reduces the burden on caregivers in Germany and that may be implemented in other countries.

The level of caregiver burden was not affected by whether the patient had schizophrenia or bipolar disorder in this survey. Previous studies of the two have shown differences, [30, 31] which implies that agitation itself may confer a similar burden across the conditions. Of note, IEQ differed with the type of patient behaviours reported by caregivers during an episode of agitation with hostility and a lack of control being associated with higher IEQ. This aligns with previous research that has identified correlations between specific behaviours and burden in bipolar disorder [21, 32]. However, here there was only a 9% difference between the behaviour with the lowest associated IEQ (over excitability) and the highest (hostility) and only 5–17% of caregivers reported violence, hostility, aggression or a lack of control as symptoms they had observed in the last 12 months (data not shown). These data suggest that all symptoms associated with agitation, including those perceived as mild manifestations which are routinely underreported, confer burden to caregivers. Therapeutic strategies that reduce these behaviours will likely benefit caregivers.

From the results of this multi-country survey, despite substantial burden, it can be concluded that caregivers play an important role in the recognition and management of agitation episodes before they become severe. It has previously been reported that a high caregiver burden can be associated with poorer patient reported outcomes in mental health disorders [33]. Therefore, caregiver support must be prioritised for patients managed in the community setting. Whilst existing evidence exploring interventions to support the caregivers of patients with mental health disorders is too inconsistent to draw clear recommendations, a meta-analysis found that therapy and educational support were useful [34]. Future studies should work to ascertain the value of psychosocial support strategies in order to mitigate the burden of agitation. However, patient-focused interventions, including behavioural and pharmacological treatments, must also be assessed to reduce caregiver burden.

Some limitations of the present study warrant consideration. The study was limited to patients and caregivers willing to complete a survey, imparting a selection bias and data that are not fully generalizable. More severely impaired patients who do not have a caregiver and who were unwilling/unable to participate in the study are not represented. For patients recruited via a patient support group, a physician confirmed diagnosis could not be confirmed; however, analysis showed no differences between those recruited through this route and those recruited via their physician. The limitation of self-reported data must be considered; however, to meet the objectives of this study, it was considered the most appropriate methodology to capture the patient’s and caregiver’s perception of agitation.

## Conclusions

In conclusion, to our knowledge, this is the first study of its kind to characterise the caregivers of schizophrenia and bipolar disorder patients who experience episodes of agitation, and the impact caregiving has on their lives. Caring for a patient who suffers with schizophrenia or bipolar disorder with episodes of agitation confers a substantial burden. Certain factors including the number of hours of care provided and patient behaviours impact this burden. Caregivers are able to recognise agitation episodes, and will intervene before an episode becomes severe. There is a need for effective interventions to support them in order to alleviate this burden.

## References

[1] Lindenmayer JP (2000). The pathophysiology of agitation. J Clin Psychiatry.

[2] Cummings J, Mintzer J, Brodaty H, Sano M, Banerjee S, Devanand DP (2015). Agitation in cognitive disorders: International Psychogeriatric Association provisional consensus clinical and research definition. Int Psychogeriatr.

[3] Nordstrom K, Allen MH (2007). Managing the acutely agitated and psychotic patient. CNS Spectr.

[4] Garriga M, Pacchiarotti I, Kasper S, Zeller SL, Allen MH, Vázquez G (2016). Assessment and management of agitation in psychiatry: expert consensus. World J Biol Psychiatry..

[5] Serrano-Blanco A, Rubio-Valera M, Aznar-Lou I, Baladón Higuera L, Gibert K, Gracia Canales A (2017). In-patient costs of agitation and containment in a mental health catchment area. BMC Psychiatry..

[6] Sachs GS (2006). A review of agitation in mental illness: burden of illness and underlying pathology. J Clin Psychiatry.

[7] Schleifer JJ (2011). Management of acute agitation in psychosis: an evidence-based approach in the USA. Adv Psychiatr Treat.

[8] Rubio-Valera M, Huerta-Ramos E, Baladón L, Aznar-Lou I, Ortiz-Moreno JM, Luciano JV (2016). Qualitative study of the agitation states and their characterization, and the interventions used to attend them. Actas Esp Psiquiatr..

[9] San L, Marksteiner J, Zwanzger P, Figuero MA, Romero FT, Kyropoulos G (2016). State of acute agitation at psychiatric emergencies in Europe: The STAGE Study. Clin Pract Epidemiol Ment Health CP EMH..

[10] Boudreaux ED, Allen MH, Claassen C, Currier GW, Bertman L, Glick R (2009). The psychiatric emergency research collaboration-01: methods and results. Gen Hosp Psychiatry.

[11] Knapp M, McDaid D, Mossialos E, Thornicroft G. Mental health policy and practice across Europe. World Health Organization. 2007. http://www.euro.who.int/__data/assets/pdf_file/0007/96451/E89814.pdf. Accessed 8 Sept 2017.

[12] Knapp M, Chisholm D, Astin J, Lelliott P, Audini B (1997). The cost consequences of changing the hospital–community balance: the mental health residential care study. Psychol Med.

[13] Adamou M (2005). Community service models for schizophrenia: evidence-based implications and future directions. Psychiatry Edgmont..

[14] Jan Shah A, Wadoo O (2010). Psychological distress in carers of people with mental disorders. Br J Med Pract..

[15] Berglund E, Lytsy P, Westerling R (2015). Health and wellbeing in informal caregivers and non-caregivers: a comparative cross-sectional study of the Swedish general population. Health Qual Life Outcomes..

[16] Flyckt L, Löthman A, Jörgensen L, Rylander A, Koernig T (2013). Burden of informal care giving to patients with psychoses: a descriptive and methodological study. Int J Soc Psychiatry.

[17] Awad AG, Voruganti LNP (2008). The burden of schizophrenia on caregivers: a review. PharmacoEconomics..

[18] Ogilvie AD, Morant N, Goodwin GM (2005). The burden on informal caregivers of people with bipolar disorder. Bipolar Disord.

[19] Van Der Voort TYG, Goossens PJJ, Van Der Bijl JJ (2007). Burden, coping and needs for support of caregivers for patients with a bipolar disorder: a systematic review. J Psychiatr Ment Health Nurs.

[20] Yazici E, Karabulut Ü, Yildiz M, Baskan Tejes S, İnan E, Çakir U (2016). Burden on caregivers of patients with schizophrenia and related factors. Nöro Psikiyatri Arş..

[21] Perlick DA, Rosenheck RA, Miklowitz DJ, Chessick C, Wolff N, Kaczynski R (2007). Prevalence and correlates of burden among caregivers of patients with bipolar disorder enrolled in the Systematic Treatment Enhancement Program for Bipolar Disorder. Bipolar Disord.

[22] Goossens PJJ, Van Wijngaarden B, Knoppert-van Der Klein EAM, Van Achterberg T (2008). Family caregiving in bipolar disorder: caregiver consequences, caregiver coping styles, and caregiver distress. Int J Soc Psychiatry.

[23] Roick C, Heider D, Bebbington PE, Angermeyer MC, Azorin J-M, Brugha TS (2007). Burden on caregivers of people with schizophrenia: comparison between Germany and Britain. Br J Psychiatry J Ment Sci..

[24] Perlick D, Clarkin JF, Sirey J, Raue P, Greenfield S, Struening E (1999). Burden experienced by care-givers of persons with bipolar affective disorder. Br J Psychiatry J Ment Sci..

[25] van Wijngaarden B, Schene AH, Koeter M, Vázquez-Barquero JL, Knudsen HC, Lasalvia A (2000). Caregiving in schizophrenia: development, internal consistency and reliability of the Involvement Evaluation Questionnaire-European version. Br J Psychiatry.

[26] Pompili M, Harnic D, Gonda X, Forte A, Dominici G, Innamorati M (2014). Impact of living with bipolar patients: making sense of caregivers’ burden. World J Psychiatry..

[27] Hirst M (2005). Carer distress: a prospective, population-based study. Soc Sci Med.

[28] Swain SP, Behura SS, Dash MK (2017). A comparative study of family burden and quality of life between caregivers of schizophrenia and dementia patients. Int J Community Med Public Health..

[29] von Kardorff E, Soltaninejad A, Kamali M, Shahrbabaki ME (2016). Family caregiver burden in mental illnesses: the case of affective disorders and schizophrenia—a qualitative exploratory study. Nord J Psychiatry.

[30] Grover S, Chakrabarti S, Aggarwal M, Avasthi A, Kulhara P, Sharma S (2012). Comparative study of the experience of caregiving in bipolar affective disorder and schizophrenia. Int J Soc Psychiatry.

[31] Vasudeva S, Sekhar CK, Rao PG (2013). Caregivers burden of patients with schizophrenia and bipolar disorder: a sectional study. Indian J Psychol Med..

[32] Zhou Y, Rosenheck R, Mohamed S, Ou Y, Ning Y, He H (2016). Comparison of burden among family members of patients diagnosed with schizophrenia and bipolar disorder in a large acute psychiatric hospital in China. BMC Psychiatry..

[33] Perlick DA, Rosenheck RR, Clarkin JF, Raue P, Sirey J (2001). Impact of family burden and patient symptom status on clinical outcome in bipolar affective disorder. J Nerv Ment Dis..

[34] Yesufu-Udechuku A, Harrison B, Mayo-Wilson E, Young N, Woodhams P, Shiers D (2015). Interventions to improve the experience of caring for people with severe mental illness: systematic review and meta-analysis. Br J Psychiatry.

